# Colonic Fluid and Electrolyte Transport 2022: An Update

**DOI:** 10.3390/cells11101712

**Published:** 2022-05-22

**Authors:** Abel B. Negussie, Annika C. Dell, Bruce A. Davis, John P. Geibel

**Affiliations:** 1 Department of Molecular, Cellular, and Developmental Biology, Yale University, New Haven, CT 06510, USA; abel.negussie@yale.edu; 2 The John B. Pierce Laboratory, Inc., New Haven, CT 06519, USA; adell@jbpierce.org; 3 Department of Surgery, Yale University School of Medicine, New Haven, CT 06520, USA; bruce.davis@yale.edu; 4 Department of Cellular and Molecular Physiology, Yale University School of Medicine, New Haven, CT 06520, USA

**Keywords:** colon physiology, colonic ion transport, CFTR, ENaC, H,KATPase, NHE, NKCC, IBS

## Abstract

Colonic epithelial cells are responsible for maintaining a delicate balance between luminal secretion and the absorption of fluids and ions. This review aims to discuss and update the model of colonic electrolyte secretion and absorption via the cystic fibrosis transmembrane regulator (CFTR), epithelial sodium channel (ENaC), Na-K-Cl cotransporters (NKCC1 and 2), Na-H exchangers (NHE1–4), colonic H,KATPase, and several other key components involved in multi-level transepithelial ion transport. Developments in our understanding of the activity, regulation, localization, and relationships of these ion transporters and their interactions have helped forge a more robust understanding of colonic ion movement that accounts for the colonic epithelium’s role in mucosal pH modulation, the setting of osmotic gradients pivotal for fluid retention and secretion, and cell death regulation. Deviations from homeostatic ion transport cause diarrhea, constipation, and epithelial cell death and contribute to cystic fibrosis, irritable bowel syndrome (IBS), ulcerative colitis, and cancer pathologies. Signal transduction pathways that regulate electrolyte movement and the regulatory relationships between various sensors and transporters (CFTR as a target of CaSR regulation and as a regulator of ENaC and DRA, for example) are imperative aspects of a dynamic and comprehensive model of colonic ion homeostasis.

## 1. Introduction

**Figure 1:**
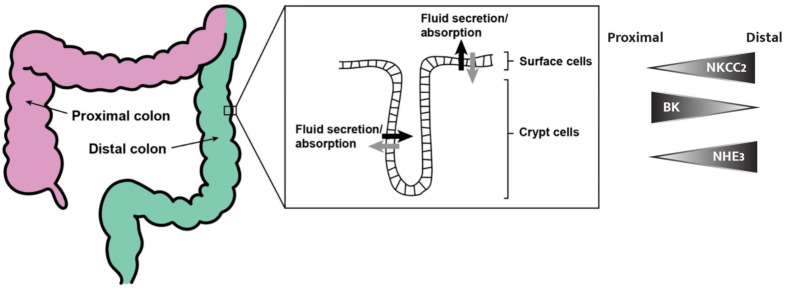
Schematic model of the colon and crypt. We refer to everything from the cecum to the left colic flexure as the proximal colon (pink) and everything spanning from the proximal colon to the perianal junction as the distal colon (green). An expansion of the luminal colonic epithelium (right) shows the crypt–surface cell axis and a model for fluid secretion and absorption, where both cell types secrete and absorb fluids and electrolytes [4]. The right panel shows an intensity profile graphic representing the distribution of NKCC2, BK channels, and NHE3 (transport proteins described in more detail later in this review) from the proximal to distal colon.

The colon is the final segment of the vertebrate digestive system, where fluid and electrolyte transport can be modulated to maintain intestinal and whole-body homeostasis. It is important that we understand the mechanisms through which secretion and absorption occur, and how these pathways are involved in health and disease. The expression and activity of many ion transporters in the colon are controlled by a complex and delicate homeostatic ion balance, such that hormones (i.e., aldosterone and angiotensin), pathophysiological inhibitors (i.e., Cholera and STa toxins) and diseases (i.e., metastatic changes) prompt surprisingly distinct responses between the proximal and distal colon segments (Figure 1) [1,2,3]. Modern and classical experimental methods, such as real-time reverse transcription polymerase chain reaction (RT-PCR), Ussing chambers, genome-wide analysis, next-generation epigenetic sequencing analysis, immunocytochemistry, patch clamping, and siRNA, have allowed the localization, characterization, and measurement of ion transporters. The differential expression and regulation of transporters, described using the following methods, is what gives the proximal and distal colon, apical and basolateral epithelial membranes, and crypts and surface cells unique homeostatic functions and responses to various drugs, hormones, and immune factors [1,2,3].

### 1.1. Electrophysiology

Ion transporters on the basolateral and apical membranes of colonic epithelial cells work in parallel to allow transepithelial ion absorption and secretion while maintaining a homeostatic intracellular ionic milieu. Historically, the Ussing chamber, which uses short-circuit current to determine net transepithelial ion movement properties, was the first means of measuring ionic flux. In an Ussing chamber, both sides of the epithelium are typically flanked by identical electrolyte solutions to eliminate possible paracellular ion movement down osmotic or electrostatic gradients. Since the 1950s, Ussing chambers and derivatives of the technology have been used to observe homeostatic, disease state, and drug- and hormone-induced transepithelial ion movement properties of the colon and countless other mammalian tissues [5]. Clarke offers a comprehensive overview of the Ussing chamber methodology and its applications [5].

### 1.2. Isotopic Flux

In addition to short-circuit currents, the radioactive labeling of electrolyte solutions emerged as another method to measure transepithelial ion movement in Ussing chambers. Radioactive flux studies begin with an electrolyte solution isolated from its isotopic solution by an epithelium; the nature of the epithelium’s ionic transport capabilities is determined by changes in isotope concentrations in the chambers over time, which is indicative of an ionic flux [5,6].

### 1.3. Intracellular Ion Activity and Transporter Measurements

Measuring an ion current through a specific transporter or channel is also possible using the patch clamp and vibrating probe methods [7]. With patch clamping, epithelial cells of interest are isolated and a pipette is then attached to the cell, allowing for “encapsulation” and a detailed evaluation of a single ion channel. The changes in current that are detected via the pipette and patch clamp amplifier are recorded, and the investigator can then set an intermembrane potential and measure current changes that are occurring due to the activation or inactivation of the channel of interest in the native state, and also while adding inhibitors or activators of the channel. Patch clamping, along with the vibrating probe that measures local current densities on a membrane, is membrane-specific and has allowed a mechanistic understanding and dissection of transepithelial ion transport through the analysis of apical and basolateral membrane transporters and channels in isolation [4].

### 1.4. Isolated Perfused Crypts

Various fluorescent and radioactive screening methods that allow live monitoring of the intracellular ion concentrations, pH levels, and membrane potentials of cells in isolated colonic crypts have been developed. These methods have been essential in developing a comprehensive understanding of the many secretory and resorptive mechanisms of colonocytes when exposed to a wide variety of experimental extracellular milieu (exposure to cyclic nucleotides, antibiotics, or divalent cation nanoparticles, to name a few). Furthermore, research on isolated colonic crypts using perfusion through concentric pipettes has revealed much about transepithelial electrophysiology since the method’s development by Dr. Maurice Burg [8] and revision by Dr. Rainer Greger and his colleagues [9], as well as by Dr. John P. Geibel and his colleagues [10].

### 1.5. The Molecular Characterization

Immunocytochemistry serves as a visual assay for determining on which membrane a particular ion channel protein is expressed, while patch clamping measures conductance across one membrane to characterize and measure the activity of a channel of interest on either the apical or basolateral membrane [4,11]. Both methods have been useful in determining the mechanism of transepithelial electrolyte movement, as these processes involve the membrane-specific recruitment or localization of different ion channels. Apical and basolateral recruitment and localization are not, however, fixed; RT-PCR measurements demonstrate that ion transporter expression levels, localization, and recruitment are readily modified to account for short-term changes in water or electrolyte intake and other luminal conditions that can adversely or positively affect the homeostasis of fluids and salts [12].

Because different gene expression profiles of ion channels and regulators exist across the colonic longitudinal axis, everything from subtle segmental differences in aldosterone sensitivity to differences in the direction of net ion movement (as is the case for K^+^) have been observed between the proximal and distal colons (Figure 1) [2,13,14]. Prolactin, for instance, exhibits opposite effects on ion secretion between the proximal and distal colon [3]. Chromatin immunoprecipitation next-generation sequencing (ChIP-Seq) revealed that of 9866 actively expressed genes in the rat colon, 540 are differentially expressed between distal and proximal segments [1]. This would explain the observed site-specific colon pathology (particularly tumorigenesis), differential effects of drugs and hormones, and even different homeostatic functions of the various segments in electrolyte movement [1,2].

There are also significant differences in the gene expression profiles between crypt and surface cells. The ouabain-sensitive H,KATPase, for instance, is localized in crypt cells, while the ouabain-insensitive isoform is primarily expressed in surface cells [15]. The catechol-O-methyltransferase (COMT) enzyme is another enzyme that is particularly concentrated in the apex of crypts [16]. Perhaps the most significant difference between the two relates to the roles they play in facilitating epithelial turnover during extrusion. The base of a crypt exhibits high proliferation and few signs of differentiation; however, proliferation decreases while expression of differentiation markers increases when moving towards the surface [4]. There continues to be some confusion in the literature on the distinction between crypt and surface cells regarding their respective roles in secretion and absorption [4,17,18]. Several patch clamp experiments have already demonstrated that neither secretion nor absorption are exclusive to the crypts or surface cells [4]. It is the dismantling of this misconception that has allowed models that can explain the large volumes of fluid secretion possible in the colon, particularly under disease states such as secretory diarrhea, with the apical surface area of the epithelium contributing to the loss of electrolytes and the associated fluid movement from cells to the lumen of the colon [4]. This review aims to briefly describe the homeostatic function of the major ion transporters in the colon and to explain recent developments in understanding the ion transport mechanisms of colonic pathologies and the associated cell death due to destruction of the homeostasis found in the normal physiology of the colon.

## 2. Secretion

Secretion is essential to the homeostatic function of the colon and is of great interest because it is the mechanism responsible for the most prevalent forms of diarrhea. The colon is the body’s final secretory segment where transepithelial secretion is achieved by concurrent apical efflux of electrolytes through CFTR and BK channels, along with basolateral electrolyte influx through the sodium–potassium–chloride cotransporters (NKCCs) and other transporters and channels described later in this review. These transmembrane proteins, along with small amounts of paracellular flux, are responsible for extrusion of salt into the lumen. Proper functioning of these systems is essential to maintain a healthy intracellular ionic milieu. The processes involved in colonic ion secretion also allow for appropriate stool hydration and the excretion of excess salt and fluid. Deviations from normal secretory function can have dramatic effects on whole-body homeostasis; interruption of secretory pathways through activation of the calcium sensing receptor (CaSR) by Ca^2+^, for example, leads to constipation [19]. Here, we will describe the molecular and cellular characterizations of some of the best-understood transmembrane proteins involved in fluid and electrolyte secretion.

### 2.1. Paracellular Transport

**Figure 2:**
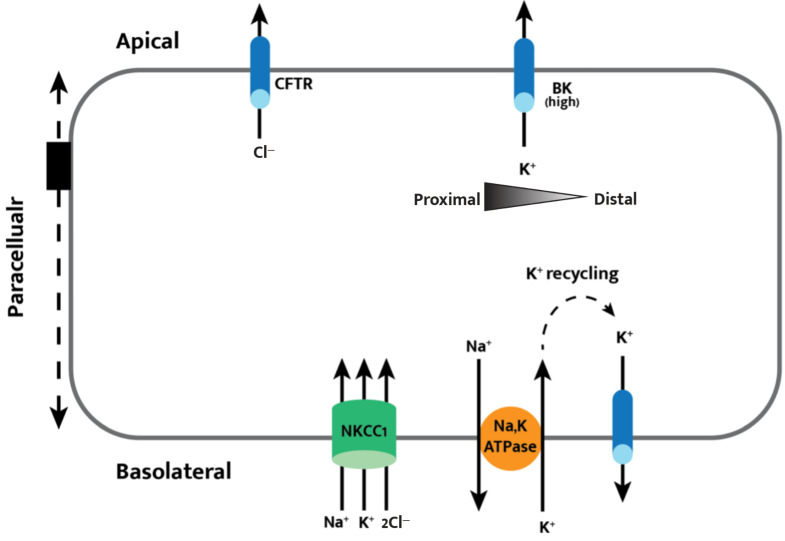
Cell model of transepithelial chloride secretion. Basolateral intake through NKCC1 of sodium, potassium, and chloride allow for apical secretion of chloride through CFTR. Apical BK channel expression is higher in the proximal colon, leading to an observed net potassium secretion in the proximal colon and net potassium absorption in the distal colon. The apical and basolateral membranes are functionally coupled by the paracellular pathway, acting as a potential shunt for ions and fluid that is essential in secretory epithelia.

The epithelium of the large and small intestine has a low electrical resistance when compared to skin or other organs [20]. This highly electric permeability allows for the necessary task of facilitating the paracellular transport of water, ions, and nutrients while providing a barrier to larger molecules and microbes that reside in the gut lumen [20]. Secretory epithelia are polarized layers of cells that require distinct sets of apical and basolateral membrane transporters to allow for ion transport, as seen in Figure 2. The apical and basolateral membranes are functionally coupled by the paracellular pathway [20]. This paracellular transport, referring to the passage of molecules between adjacent epithelial cells, for ions and fluid is essential in secretory epithelia in the colon. The paracellular pathway facilitates secretion in the colon by inducing hyperpolarization of the apical membrane away from the Cl^−^ equilibrium potential (ECl) during stimulation of apical Cl^−^ channels and supports apical driving force for Cl^−^ secretion [21].

### 2.2. Sodium–Potassium–Chloride Cotransporter Type 1 (NKCC1)

To maintain homeostatic intracellular Cl^−^ concentrations while also achieving transepithelial chloride secretion, chloride must be absorbed from the basolateral membrane in parallel with apical secretion by CFTR (Figure 2) [4,14,17]. NKCC1 was first described upon observing cDNA isolated from shark tissue in 1994 [22]; shortly after, the human homologue of the protein was found to be 1212 amino acids long, have 12 transmembrane segments, and aid transcellular movement of chloride by facilitating electroneutral ion transport with a 1 Na^+^, 1 K^+^, and 2 Cl^−^ stoichiometry [23].

NKCC1 enables transepithelial Cl^−^ and K^+^ secretion by allowing Na^+^, K^+^, and Cl^−^ into crypt and surface cells on the basolateral membrane. NKCC1 is upregulated during increased apical efflux of chloride through an intracellular Cl^−^ sensing mechanism that ensures a homeostatic electrochemical equilibrium of Cl^−^ in epithelial cells [4]. NKCC1 also provides intracellular potassium essential for K^+^ secretion through apical potassium channels, as demonstrated by decreased capacity for potassium secretion by the apical surface in the presence of the NKCC1 inhibitor bumetanide [24,25]. This model of basolateral NKCC1 cotransport facilitating chloride and potassium extrusion follows a common theme of basolateral and apical ion transporters working in unison to accomplish transepithelial ion secretion or absorption.

### 2.3. Cystic Fibrosis Transmembrane Conductance Regulator (CFTR)

The gene encoding the dysfunctional mutant CFTR chloride channel (the genetic basis for cystic fibrosis) was “the first disease gene to be isolated without the use of previous cytogenic clues” during the inception of the human genome project [26]. The CFTR protein is an ABC-transporter-class ion channel with two symmetrical sides, each with six membrane spanning domains and one cytoplasmic ATP binding site, connected by a cytoplasmic regulatory “R” domain in between which opens the channel to Cl^−^ and HCO_3_^−^ efflux when ATP is bound, while the R domain is phosphorylated by PKA [27]. Coupled with chloride intake from the basolateral membrane by NKCC1 is Cl^−^ efflux across the apical membrane; CFTR is known as the main source for the apical secretion of Cl^−^ in the colon and plays a major role in direct transepithelial secretion [28]. CFTR is also involved in the regulation of absorption through the inhibition of the epithelial Na^+^ channel (ENaC) and is required for cAMP stimulation of DRA (read the absorption section for more on ENaC and DRA) [28,29]. CFTR knockout mice exhibit major colonic dysfunction as a result of inadequate Cl^−^ efflux that cannot be compensated for by hypersecretion by other apical chloride channels such as anion exchange (AE) and downregulated in adenoma (DRA) [4,28]. The pathology resulting from an inactive CFTR can be attributed to the reduction in chloride secretion that is involved in the normal movement of stools and enzymatic digestion in the colon [30]. In fact, chloride secretion is the primary driving force for the osmotic diffusion of fluids into the lumen of the colon [30]. CFTR has been described as a gatekeeper of osmotic flow in the colon’s epithelium. For this reason, the regulators and signaling pathways of CTFR have been examined in detail to better understand the fluid-secretion-related pathology. Anderson et al. offer a comprehensive overview of CFTR’s implication in colorectal cancer [27].

CFTR is activated by cAMP-, cGMP-, and Ca^2+^-dependent phosphorylation by classic second messenger systems, including PKA [28]. A few mechanisms have been explored with respect to how increased cytoplasmic cAMP upregulates CFTR activity; cAMP increases CFTR localization in the apical membrane via either increased exocytosis or decreased endocytosis of CFTR-containing vesicles, and via movement of the transporter from the cytoplasm to the apical plasma membrane [28,31]. Similar pathways exist for the cGMP-dependent activation of CFTR [28]. Although the Ca^2+^-dependent pathways for CFTR activation are less understood, models of PKA-, cAMP-, and cGMP-dependent inter-regulatory pathways have been proposed and continue to be explored, as there is a high interest in CFTR regulatory pathways as potential therapeutic targets for secretory diseases [4,32,33]. This research on regulatory pathways for CFTR and their effectors will be discussed in further detail later in this review.

### 2.4. K^+^ Secretion

Net K^+^ secretion is only observed in the proximal colon, along with net absorption in the distal colon [11,14]. The opposite segmental functionality of the proximal and distal colon with regard to potassium ion movement can potentially be explained by the increased expression of the potassium–calcium-activated channel subfamily M alpha 1 (KCNMA1 or BK channels) in the proximal colon (Figure 2). In the proximal colon, 39% of surface colonocyte patches contain the channels as opposed to 12% of patches in the distal colon [11]. BK channels are large conductance potassium channels that are activated by intracellular cAMP and Ca^2+^ [14,15]. They are the most well-understood potassium channels known to be expressed and localized to the apical membrane (determined by RT-PCR and immunolocalization, respectively), and they are also necessary for K^+^ secretion. BK knockout mice lack the ability to secrete K^+^ and exhibit low fecal potassium levels [14,34]. The simplified and standard model for potassium secretion includes BK channels working in parallel with basolateral NKCC1 and Na,KATPase to facilitate active transepithelial K^+^ secretion. There is evidence, however, for a Ba^2+^-sensitive renal outer medullary potassium (ROMK) channel in the apical membrane of colonic epithelial cells [35]. More research is needed to better understand potassium conductance through the ROMK channel, as its apical localization is contradictory to more recent findings that suggest BK and intermediate channel exclusivity [15]. Intermediate conductance K^+^ (IK) channels have also been localized by immunofluorescence to both the apical and basolateral membranes of colonic epithelial cells. Because IK knockout mice exhibit increased K^+^ secretory activity, it has been suggested that IK channels serve to recycle K^+^ on the basolateral membrane under basal conditions. On the apical membrane, K efflux through IK channels is a driving force for Cl^−^ secretion [15].

NKCC1 (described above) and the Na,KATPase, which catalyze the exchange of 3 Na^+^ ions for 2K^+^ ions, are the main means of basolateral potassium uptake. The electrochemical gradient generated by the Na,KATPase (low intracellular Na^+^, high intracellular K^+^, and a membrane potential of around −50 mV) provides the driving force for apical Na^+^-absorptive processes, including NAH- and ENaC-mediated transport (described in Section 3) [15,35,36]. The ATPase facilitates active electrogenic potassium uptake in both the proximal and distal colon, although only to produce net K^+^ secretion in the proximal colon, at least in part due to the unequal distribution of apical BK channels described previously [11]. Because the Na,KATPase activity is stimulated by aldosterone, its administration converts the distal colon to also exhibit net potassium secretion [36]. Furthermore, potassium secretion can be inhibited in the descending colon using the cardiac glycoside drug ouabain due to its ability to inhibit the Na,KATPase [36]. These experiments emphasize the importance of the ATPase in modulating potassium secretion [35,36]. Rajendran and Sandle offer a comprehensive description of colonic K^+^ absorption and secretion mechanisms and a characterization of important diseases with respect to K^+^ balance [15].

## 3. Absorption

Electrolyte homeostasis and osmotic gradients across the colon’s epithelium depend on a balance between transepithelial luminal secretion and both electroneutral and electrogenic absorption of NaCl, K^+^, and Cl^−^ [4]. Special attention must be given to the proximal–distal distribution and the up- and downregulation of key transporters involved in absorption that are known to be sensitive to hormonal status and epigenetic modification [37,38]. For instance, net Na^+^ absorption tends to decrease from the proximal to distal colon [37] and amiloride-sensitive Na^+^ channels are the primary mode of absorption under high aldosterone conditions [4].

To summarize the colon’s ion absorption functionality, we will first discuss essential regulators of intracellular pH, including Na-H exchanger (NHE) and the colonic H,KATPase, as well as other apical mechanisms of electroneutral absorption. Next, we will describe the complex multilevel coordination that colonocytes use to ensure the parallel regulation of apical absorption (through ENaC) and basolateral exit of Na^+^ (through the Na,KATPase) to maintain a healthy intracellular milieu.

### 3.1. Electroneutral Absorption

#### 3.1.1. Sodium–Potassium–Chloride Cotransporter 2 (NKCC2)

Sodium–potassium–chloride cotransporter 2 is another NKCC isoform that has recently been shown to localize in the apical membrane of distal colonic epithelial cells (Figure 1), catalyzing the electroneutral absorption of Cl^−^, K^+^, and Na^+^. Unlike its basolateral counterpart (NKCC1), NKCC2 contributes to chloride absorption and demonstrates another pathway for Cl^−^ uptake other than through the commonly attributed Cl/HCO_3_^−^ and Cl/OH^−^ anion exchange processes [39,40]. The importance of NKCC2 in regulating fluid retention has been demonstrated by Western blotting experiments, where a 90% increase in NKCC2 protein expression levels in the distal colon was observed after a regiment of low water intake by mice. Furthermore, short-term stimulation of colonocytes by vasopressin was found to triple NKCC2 phosphorylation, likely through cAMP-PKA- and Ca^2+^-mediated signaling pathways [39,40]. These findings suggest that NKCC2 plays an important role in retaining water, particularly under a dehydrated state, and makes NKCC2 and its activation pathways of interest as therapeutic targets for secretory diseases.

#### 3.1.2. Na-H Exchangers (NHE)

Two domains of a human NHE were first described by Sardet in 1989 [41]. The transmembrane domain consists of twelve membrane-spanning domains comprised of around 500 amino acids in total and is responsible for the electroneutral exchange of intracellular protons for extracellular sodium through the fourth and fifth transmembrane regions (TM4 and TM5) with a 1Na^+^:1H^+^ stoichiometry. The C-terminus cytoplasmic tail domain is responsible for the exchanger’s sensitivity to growth factors, lipid signaling, and phosphorylation [41,42,43]. Isolated colonic crypts exhibit rapid (or non-genomic) responses to aldosterone, including increases in NHE activity in human and rat colonic crypts, although the exact mechanism is unknown [44]. Cl^−^-dependent NHE activity has also been observed in crypt cells and likely occurs through the coupling of a chloride channel (potentially CFTR) to NHE [45]. Cl^−^-dependent NHE activity is critical for the proper regulation and recovery of intracellular pH, a key function of Na-H exchange [45].

To date, ten distinct isoforms of Na-H exchangers have been identified in human tissues (NHE1–10), although their structures are highly conserved; we will focus here on isoforms that are best understood and most relevant to colon physiology. NHE1, known as the “housekeeping” isoform due to its universal role in maintaining basic cellular pH and volume homeostasis, is expressed on the basolateral membrane in colonocytes and ubiquitously expressed in other human tissues [42]. NHE1 protects renal tubule epithelial cells from acidosis through a negative feedback loop, where intracellular acid allosterically activates the exchanger. NHE1 also protects renal tubule epithelial cells from osmotic shrinkage through two known mechanisms when exposed to a hypertonic extracellular environment. First, absorption of sodium is coupled with secretion of acid which can be replenished by dissociation of intracellular weak acids, creating a net increase in intracellular osmolality. Second, the epithelial cell membrane is more permeable to carbon dioxide at higher intracellular pH’s, meaning Na-H exchange activity provides increased intracellular bicarbonate, which drives increased Cl^−^/HCO_3_^−^ exchange (increased absorption of chloride) [42]. Net NaCl absorption, therefore, is a result of Na-H activity, which causes an osmotic gradient that leads to the entrance of water through aquaporins, including AQP3, which is the predominant aquaporin isoform expressed in the human colon [46]. Interest in aquaporins has recently grown due to new findings suggesting that they play important roles in cell volume regulation, diarrhea, irritable bowel syndrome, and colorectal cancer pathology, which we will discuss in Section 4.

**Figure 3:**
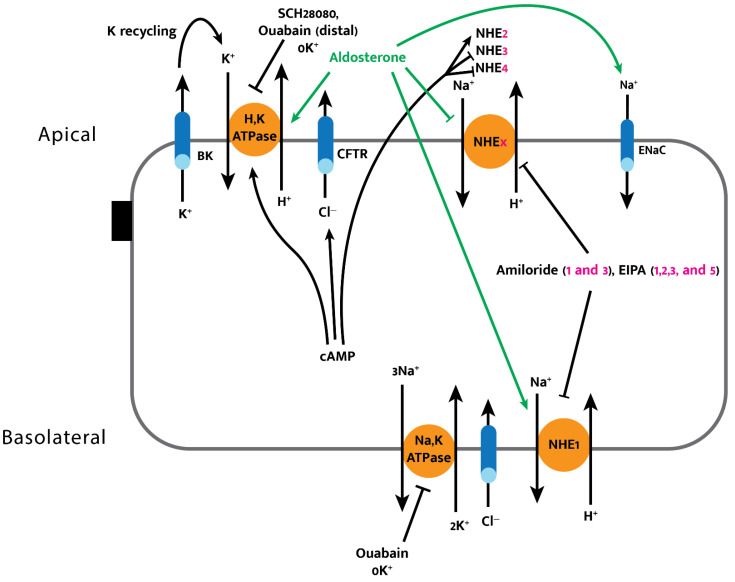
Hormone- and cAMP-mediated regulation of colonic electrolyte movement. Aldosterone inhibits apical NHE isoforms while activating H,KATPase, ENaC, and basolateral NHE1 in the distal colon; cAMP activates H,KATPase and NHE2 while inhibiting NHE3 and 4. The specific NHE isoforms modulated by amiloride and its analog EIPA are listed in parentheses. Ouabain exhibits an inhibitory effect on the Na,KATPase and on the apical H,KATPase in the distal colon.

The uptake of luminal sodium by the NHE2 and NHE3 isoforms, which are localized on the apical membrane of colonic epithelial cells, is tightly coupled with Cl^−^/HCO_3_^−^ exchange to ultimately achieve NaCl absorption in both crypt and surface cells, although only the distal colon exhibits downregulation of these isoforms under Na^+^ depletion (in the presence of aldosterone) (Figure 3) [4,43]. NHE3 is also expressed on endosomes, where it helps facilitate early endosome acidification [43]. Recently, intestinal epithelial cell-specific NHE3 knockout mouse models (NHE3IEC-KO) were generated to better understand NHE3 and the role it plays with regard to Na^+^ and fluid absorption in vivo [47]. Xue et al. (2020) demonstrated that intestinal NHE3 contributes to acid–base, Na^+^, and volume homeostasis, and that a lack of intestinal NHE3 has consequences for intestinal structural integrity, as NHE3IEC-KO mice presented with diarrhea, swollen intestines, and increased intestinal permeability [47].

The NHE1 and NHE3 isoforms are inhibited by amiloride with varying inhibitory potencies (IC_50_ = 5.3 and 100–309 μM, respectively, for rat NHE1 and -3) via amiloride exposure [48]. Furthermore, the amiloride derivative ethylisopropylamiloride (EIPA) can inhibit NHE1, -2, -3, and -5 (ordered by increasing inhibitor binding affinity) at small concentrations [48]. The recently characterized NHE4 isoform, however, is characteristically resistant to amiloride and EIPA, making selective inhibition an easy way to specifically study NHE4 activity [44,48]. The perfusion of isolated human and rat colonic crypts loaded with pH-sensitive fluorescent dye (BCECF) allows for real-time intracellular pH measurements that provide insights into sodium-dependent proton exchange during exposure to hormones, drugs, secondary messengers, and various extracellular ionic milieu [44]. These experiments have shown that NHE4, like NHE1, plays a role in pH and cell volume regulation in the human colonic crypt, and that this isoform, along with NHE3, is downregulated by cAMP while NHE1 and NHE2 are upregulated by cAMP exposure [44].

Aldosterone’s effects are exemplary of the complex multi-level feedback and parallel regulation of apical and basolateral compartments required to precisely modulate electrolyte levels. In the mature distal colon, aldosterone stimulates apical ENaC and basolateral Na,KATPase, while inhibiting apical NHE3 activity. These effects facilitate a switch from electroneutral to electrogenic Na^+^ absorption [49,50]. Apical NHE and H,KATPase are the only known mechanisms for acid extrusion by the colonic epithelium; aldosterone maintains homeostatic H^+^ secretion despite the reduction in apical NHE activity by simultaneously upregulating the colonic H,KATPase (described below) [49,50].

#### 3.1.3. Colonic H,KATPase

The non-gastric or colonic H,KATPase is a P2-type heterodimeric ATPase that facilitates the active electroneutral exchange of extracellular K^+^ for cytoplasmic H^+^ ions. Immunocytochemistry, RT-PCR, and sequence cloning have been utilized to determine both the presence of the H,KATPase in the colonic epithelial cells, as well to determine some of its anti-apoptotic properties [12]. Although a beta subunit specific to the colonic H,KATPase has not been identified, the alpha subunit, HKalpha2, is unique to the colon and contains the transmembrane domain responsible for ion translocation. The colonic H,KATPase is structurally similar to the previously described basolateral Na,KATPase; in fact, the beta subunit of either pump can dimerize with HKalpha2 to effectively catalyze active proton–potassium exchange [12]. Aldosterone has been shown to have an upregulating effect on HKalpha2 expression in the rat distal colon (where net potassium absorption is observed) [15]. The ouabain sensitivity of H,KATPase activity differs across the crypt–surface axis in the rat distal colon, with ouabain inhibition is only observed in crypt cells. It is hypothesized that two distinct beta subunits for the colonic H,KATPase are responsible for differential ouabain sensitivity [15].

Despite the colon’s ability to precisely modulate potassium levels, passive diffusion is often, and likely incorrectly, credited for apical K^+^ uptake by the colon [15,36]. Most evidence points rather to active K^+^ absorption mediated by the colonic H,KATPase. HKalpha2 homozygous knockout mice exhibit high fecal losses of potassium, low levels of intracellular potassium, loss of body weight, and decreased ENaC-mediated Na^+^ absorption [15]. Apical absorption of K^+^ by H,KATPase is part of a transepithelial potassium absorption pathway because it is coupled with the electroneutral exit of K^+^ across the basolateral membrane through Ca^2+^-activated and cAMP-activated potassium channels [15].

Colonocytes undergo regulatory volume increases after hyperosmotic shrinkage to prevent cell death. The inhibition of H,KATPase by the specific K-competitive inhibitor SCH28080 interrupts this regulatory function and makes cells more susceptible to apoptotic volume decrease, acidosis, and loss of cellular K^+^ [12]. These findings suggest that the H,KATPase counteracts these early apoptotic events, making the pump a potential therapeutic target for the treatment of malignancies through the induction of apoptosis.

#### 3.1.4. Cl^−^–HCO_3_^−^ Exchange

The Cl^−^–HCO_3_^−^ exchanger DRA (downregulated in adenoma), encoded by the SLC26A3 gene, catalyzes the electroneutral exchange of intracellular bicarbonate for Cl^−^ on the apical membrane in a manner that is closely coupled with apical Na-H exchange to facilitate NaCl absorption [4,51]. Although the exact mechanism of coupled NaCl absorption through DRA and NHE3 activity is not known, direct physical linkages between the exchangers and indirect linkages through either a multi-protein complex, local pH gradient effects, cell volume effects, or cytoskeletal association have all been proposed as potential coupling mechanisms [43].

NHE and Cl^−^/HCO_3_^−^ activity is not, however, exclusively coupled. In fact, NHE3 and DRA expression is so longitudinally segregated that only the late middle third of the colon expresses both proteins, with NHE3 expression being highest in the early proximal colon and DRA expression being highest in the late middle colon and cecum (Figure 1) [51]. Because exchange coupling can only occur when these proteins are in close proximity, this segregation results in uncoupled exchange, which generates alkaline and acidic mucosal pH levels in the cecum and early proximal colon, respectively [51].

**Figure 4:**
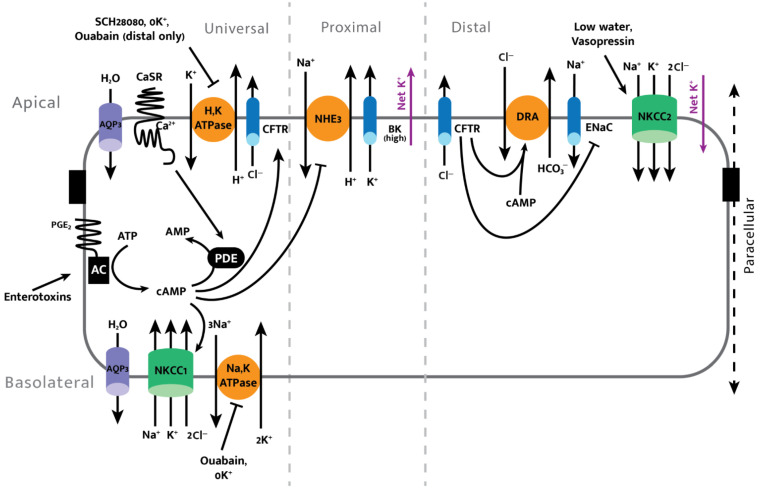
Summary of enterotoxin-induced secretion and other key interactions for colon pathophysiology. AC = adenosine cyclase; PDE = phosphodiesterase.

DRA activity is inhibited by CFTR in a cAMP-mediated process (Figure 4). There is also evidence that bicarbonate is directly transported through CFTR, meaning there is both a direct and indirect regulation of bicarbonate movement by CFTR [52].

### 3.2. Electrogenic Absorption

The epithelial Na^+^ channel (ENaC) facilitates the electrogenic uptake of sodium in the distal colon. Amiloride and its analogues inhibit ENaC, as does CFTR, explaining the excessive sodium absorption in cystic fibrosis patients [4,28]. Apical electrogenic Na^+^ absorption through ENaC is thought to occur in parallel with transepithelial Cl^−^ absorption and basolateral Na,KATPase activity to achieve net NaCl absorption and help set the osmotic gradient for water uptake. The passive absorption of sodium through both ENaC and NHE is driven by the chemical gradient established by Na,KATPase activity and the cells’ negative membrane potential [15,35,36]. ENaC is upregulated by aldosterone, with the strongest regulatory response being observed in the late distal colon (Figure 3) [13]; the aldosterone-mediated upregulation of Na^+^ absorption through ENaC induces parallel enhancement of basolateral sodium exit through the Na,KATPase through a multilevel feedback mechanism, demonstrating the complex coordination involved in homeostatic transepithelial sodium absorption [4].

## 4. Disease States

### 4.1. Diarrhea

Given the importance and scale of the colon’s secretory capacity, it is no surprise that deviations from homeostatic electrolyte balance can cause severe pathologic states, including diarrhea. Although diarrhea can be caused by several pathogens, genetic disorders, drugs, and inflammatory processes, most diarrhea types can be characterized by the increased secretion of Cl^−^ through the stimulation of CFTR- and Ca^2+^-activated Cl^−^ channels and via decreased absorption of Na^+^ through NHE3 [53]. Secretory diarrhea, which is the leading cause of death among children under the age of five worldwide, occurs when an excessive number of electrolytes is lost, subsequently followed by loss of water. Diarrhea can also be caused by impaired absorption of electrolytes (osmotic diarrhea), breakdown of the colon’s epithelium (inflammatory diarrhea), or an overly active enteric nervous system (ENS) [19].

Cholera toxins and the heat stable enterotoxin A (STa) produced by *E. coli* increase intracellular cAMP and cGMP concentrations, respectively. Cyclic nucleotides subsequently activate CFTR, DRA, and NKCC1, driving hypersecretion of Cl^−^ and bicarbonate while also inactivating NHE3, leading to less absorption of salt (Figure 4) [4]. Many bacterial and viral toxins share similar or identical cyclic-nucleotide-induced hypersecretion mechanisms, as observed in *E. coli* and cholera, making cyclic nucleotide signaling pathways promising potential targets for therapeutic solutions to secretory diarrhea [54]. Invasive bacteria, including *Salmonella* and *Shigella*, and rotaviruses tend to cause diarrhea by increasing intracellular Ca^2+^, which stimulates CFTR while inhibiting NHE3 [53]. During secretory diarrhea, the hypersecretion of chloride and bicarbonate (by activation of CFTR and DRA) along with decreased absorption of sodium due to NHE3 inhibition drive the osmotic loss of water through aquaporins (AQPs), which are water-permeable tetramer protein complexes that play a major role in fecal water content [46]. AQP3, the aquaporin most highly expressed in the colon, has also been implicated in diarrhea and constipation. Increased AQP3 expression on the apical and basal surfaces of the colonic epithelium allows more rapid equalization of transepithelial osmotic pressure gradients, leading to osmotic diarrhea or constipation, depending on the direction of the osmotic pressure gradient between the luminal and vascular sides of the colonic epithelium [46]. Osmotic laxatives such as magnesium sulfate, for instance, increase luminal osmotic pressure, to which overexpression of AQP3 is a cellular response, leading to rapid loss of water [55].

The calcium sensing receptor (CaSR) is a G-protein coupled receptor, which when activated by the luminal delivery of calcium, halts enterotoxin-induced hypersecretion and diarrhea caused by neratinib (an anticancer drug that can cause life-threatening diarrhea) [19,56,57]. The activation of CaSR by small amounts of Ca^2+^, Gd^3+^, neomycin, or other antibiotics causes a rapid increase in intracellular Ca^2+^, which promotes the PKC-facilitated destruction of cAMP and cGMP. This process ultimately reverses the secretory effect of enterotoxin-induced diarrhea and can serve as an extremely cost-effective method to treat secretory diarrhea (Figure 4) [4]. Oral calcium administered as a means to reverse secretory diarrhea via activation of the CaSR must be vitamin-D-free to prevent its early absorption in the proximal gut, thereby reducing delivered calcium to the remainder of the intestine and preventing the therapeutic effect. Because CaSR stimulation can correct the pathogenic pathways that lead to secretory, osmotic, inflammatory, and overly active ENS diarrhea, it is a general therapeutic target. The activation of AMP-activated protein kinase (AMPK) has also been shown to inhibit cholera-toxin-stimulated chloride secretion due to AMPK’s inhibitory effect on CFTR [32]. The use of systemic AMPK activators remains questionable due to off-target effects, and also since the intestine has additional chloride efflux and influx pathways. Similarly, amiloride cannot be used to treat diarrhea (via its inhibitory effects on various NHE isoforms) (Figure 3) due to its off-target systemic effects, which can be detrimental.

Excess bile acids in the colon can cause bile acid diarrhea, which is prevalent among IBS patients and in the general population, where it effects an estimated 1% of the population [58]. Bile acids increase intracellular cAMP and in turn increase chloride and water secretion (Figure 4), as well as mucosal permeability. Fibroblast growth factor 19 (FGF-19), which stimulates bile acid production, and bile acid receptors such as FXR are promising diagnostic and therapeutic targets for bile acid diarrhea [58].

Several pro-inflammatory cytokines, including IL-6, IL-1β, and interferon-γ, have been shown to induce DNA methylation, making epigenetic modulation of ion transport components a newly emerging area of interest for a deeper understanding of the pathogenesis of inflammatory bowel disease (IBD). DNA methylation in the promoter region of the gene encoding NHE3 has been shown to suppress the exchanger’s activity and to induce IBD-associated diarrhea [38]. These findings suggest epigenetic therapy as a potential treatment for IBD diarrhea.

### 4.2. Cystic Fibrosis (CF)

CF is caused by an autosomal recessive mutation in the gene encoding CFTR. The origins of the CF gene are still debated today, although a few hypotheses exist that aim to describe the prevalence of CF, which cannot be accounted for by genetic drift alone [59]. The most well-supported of these theories is that CF carriers have a heterozygote advantage of increased resistance to secretory diarrhea [59]. Because the electrochemical gradient of chloride is the largest osmotic driver of fluid secretion by colonic epithelial cells, a defect in half of expressed CFTR channels, which are the main means of apical Cl^−^ secretion, has been shown to reduce the secretory capability of the colon, and in turn reduce CF carriers’ susceptibility to secretory diarrhea as a result of cholera and *E. coli* toxins [30,60]. This theory has been refuted by skeptics of the secretory role of the colon, who claim that the CF mutation did not originate during a time where cholera was endemic in Europe, meaning the mutation could not have conferred a heterozygote advantage that would offset the decreased fitness of individuals with two copies of the CF mutation [61]. The skepticism of intestinal secretion stems from a distrust of in vitro experimental methods, such as Ussing chamber experiments, as the sole basis for the current model of intestinal and colonic chloride secretion [61]. Such skepticism has not, however, been able to: (1) explain how the intestines are able to release such large volumes of fluids during diarrheal disease if not by creating an osmotic gradient via chloride secretion; (2) address several in vivo demonstrations of chloride secretion [62,63,64,65].

### 4.3. Metastatic Disease

CFTR dysfunction and deficiency have also been heavily implicated in colorectal cancer (CRC). Low CFTR expression and the CF gene are associated with low disease-free survival in sporadic CRC and a 6 times higher risk of developing CRC, respectively [27]. CFTR deficiency as measured by low mRNA and protein expression levels, which may be caused by RAS, PKC, and IFNα signaling or epigenetic silencing, is correlated with CRC tumorigenesis and metastasis, suggesting that CFTR has an active tumor suppression role in the colon [27]. Although the mechanism of CFTR tumor suppression is not well established, several mechanisms including dysregulation of Wnt/β-catenin signaling and epithelial barrier damage induced pro-inflammatory signaling have been discovered that could explain CFTR’s tumor-suppressive role [27].

Cancer cells tend to facilitate compartmentalized concentrations of acid. Because net acid extrusion in colonic crypts is almost completely Na^+^-dependent, there is great interest in NHE activity as a means of extracellular acidification during metastatic disease [66]. NHE activity and intracellular pH are dramatically higher in cancerous human colonic crypts than in normal colon tissue, making the exchange process a prospective therapeutic target for colon cancer [66]. NHE regulatory factors (NHERFs) are also of growing interest in the study of colorectal cancer pathogenesis. Although NHERFs were originally described (and named) after their interactions with NHE3 to directly regulate apical sodium–proton exchange activity, over thirty additional protein interactions with these regulatory factors have been discovered that suggest they are functionally multifaceted and diverse [67]. The NHERF1 and NHERF2 isoforms, for example, are thought to be tumor-suppressive (by inhibiting platelet-derived growth factor signaling) and oncogenic (via suppression of the Stat3 transcription factor and CD24 gene), respectively [67].

Aquaporins also play a role in CRC pathogenesis. AQP1, AQP3, and AQP5 show elevated expression in CRC tissue in comparison to healthy surrounding tissues. For this reason, these aquaporins have been of recent interest as predictors of prognosis and as potential therapeutic targets in hopes that their association with proliferation (AQP1 and 3), metastasis (AQP1 and 3), dedifferentiation (AQP5), and drug resistance (AQP5) can be mitigated by inhibition [68]. Nagaraju et al. offer a comprehensive review of aquaporins’ roles in various GI cancers [68]. Apoptotic volume decrease (AVD) can be prevented via aquaporin inhibition by HgCl_2_, demonstrating its importance in cell volume regulation [69]. Increased cell volume (cell swelling) by aquaporin inactivation leads to cytoskeletal stretch activation of Ca^2+^ channels, leading to uncontrolled Ca^2+^ entry. Subsequent intracellular-calcium-induced PKC-facilitated destruction of cyclic nucleotides results in downregulation of CFTR and upregulation of apical NHE3 with a net effect of decreased secretion (Figure 4). Transepithelial K^+^ transport through the rat colonic epithelium is also sensitive to and involved in cell volume changes, particularly in the distal colon [70,71]; colonic epithelial cells fail to exhibit regulatory volume decrease in response to hypotonic stress when incubated in high [K^+^] conditions, illustrating the importance of a K^+^ electrochemical gradient for cell volume regulation [70]. K^+^ secretion via Ca^2+^ sensitive BK channels has been suggested as a main mechanism of cell volume regulation, including during apoptotic volume decreases [70].

### 4.4. Ulcerative Colitis (UC)

UC is a common form of IBD that involves decreased membrane integrity of the colonic mucosa, enhanced secretion, and decreased absorption of electrolytes [4,72]. Diarrhea is a common symptom of UC, and mechanisms of UC diarrhea have been explored extensively. Decreased electrogenic Na^+^ absorption and depolarization of a normally lumen-negative transmucosal potential difference in the distal colon are observed in patients with UC. Therefore, it is likely that defective sodium absorption is the primary mechanism of diarrhea in patients with UC [15]. Elevated proinflammatory cytokine levels during UC have been shown to contribute to UC diarrhea by selectively inhibiting Na^+^ absorption through ENaC [72]. Dextran sodium sulfate (DSS)-induced colitis exhibits increased expression of BK channels, and in turn net active K^+^ secretion. Furthermore, BK channels, which normally localize to surface cells and the upper portions of crypts in human and rat colons, are uniformly expressed along the crypt–surface axis in UC patients, suggesting that increased K efflux could be another contributor to UC diarrhea [15].

## 5. Conclusions

Improving our understanding of colon philology will be critical in tackling pathologies, and developing tools to do so is imperative. Organoid models of the colon are becoming increasingly important in understanding the biochemistry of the intestinal epithelium. These 3D in vitro models of the colon are becoming useful for personalized medicine for colorectal cancer and drug screening, as well as for investigating fluid, electrolyte, nutrient, and drug transport in the colon [73].

We are beginning to see the importance of the intestinal microbiome in colon philology, as well as human health and behavior. Furthermore, it has also been demonstrated that the ionic and pH environmental factors produced by colonic electrolyte transport discussed in this review shape what microbes exist and thrive in the gut [74]. This is a rapidly growing field with widespread implications for human health, where innovations in tools that measure the impact of various environments on specific bacteria will be key [74].

Being the final area that can adjust local and potentially whole-body fluid and electrolyte secretion and absorption, the colon is an important target of a variety of disease states, including diarrhea, cancer, and IBD, all of which involve dysregulation of complex ionic transport systems [4]. In this review, we set out to provide an updated overview of the major components involved in colonic fluid and electrolyte transport and their mechanisms of regulation. Due to the colon’s terminal location in the gut and its ability to be the final intestinal area to modulate secretion and absorption of salt and water, this tissue remains an important area for research and for identifying novel targets that could play an important role in the modulation of both local and systemic salt and water transport. Recently, the colonic transport proteins have been evaluated as new targets for blood pressure regulation [75] and IBS constipation [76], as the colon can modulate the body’s ability to concentrate or dilute fluid and electrolytes.

## Data Availability

Not applicable.
